# Chicago Medical Society

**Published:** 1885-03

**Authors:** 


### SOCIETY PROCEEDINGS.

CHICAGO MEDICAL SOCIETY.

*Meeting of December 15, 1884.*—The President, Dr. D. A. K. Steele, in the chair; Dr. Liston H. Montgomery, Secretary.

*Actinomycosis in the Human Subject.*—Dr. John B. Murphy read a paper on this subject, in which he first gave an interesting summary of the literature of the disease, with references to twenty-eight cases of its occurrence in the human subject, and then reported two additional cases.

*Case I.*—June 28, 1884, he was called to see Mary M., twenty-eight years old, who stated that two weeks before, she had begun to suffer with severe toothache in the left side of the lower jaw. Shortly afterwards a swelling appeared in the throat, with great pain in swallowing and inability to open the mouth. The pain and swelling disappeared after her face and neck had been poulticed for several days. A few days afterward she was again attacked with toothache, together with an indescribable ringing in her ears. A swelling appeared in her mouth, on the outer side of the jaw, the pain grew more severe, and the swelling continued to enlarge up to the time that the speaker was called. On examination, he found a swelling behind the angle of the jaw, on the left side. The patient could not open her mouth more than three-quarters of an inch, and had eaten nothing for two days. The left tonsil was much enlarged, and filled most of the pharynx; a pale-yellow spot marked the spot where an abscess was about to break. A

large quantity of pus was set free with a lancet. She made a rapid recovery, and was about in a few days. She appeared to remain comparatively well until the 8th of July, but did not regain her strength, and occasionally suffered with toothache. A small swelling now appeared on the left side of the neck, below the jaw, accompanied by some pain and inflammation, and a few days afterward she again presented herself for treatment. The lump was about as large as a walnut, and the surrounding tissue was considerably indurated. Several carious teeth were noticed in the mouth. The "bunch" fluctuated and seemed to contain considerable pus, but only a small quantity was discharged (when it was punctured) not exceeding a few drops, and having a creamy appearance. A drainage-tube was inserted and an antiseptic dressing applied. On her return, two days later, the drainage-tube was removed, and about twenty drops of thick, creamy-looking discharge oozed from the opening. It contained peculiar sulphur-colored granules, which were recognized with the aid of the microscope as actinomyces. A number of specimens were then prepared and shown to Dr. Fenger, Dr. Bridge, and Dr. Kerber, who agreed with the speaker as to their nature. The patient was under observation for ten days longer, and each day new specimens were procured. As the swelling and induration continued to increase, and as the patient was sinking rapidly, she was advised to enter the City Hospital, and Dr. W. T. Belfield was asked to examine the case. He reported that the pus contained actinomyces.

After a few days of preparatory treatment, the growth was removed, with the assistance of Dr. Fenger, Dr. Belfield, and Dr. W. P. Verity. An incision was made, extending from the ear to the clavicle, parallel with the sterno-cleido-mastoid muscle. A mass of succulent tissue was observed, interspersed with pus and gold-colored granules, penetrating the surrounding tissue. It was all removed with a knife and a sharp spoon. In some places this tissue penetrated from half to three-quarters of an inch. A carious tooth was removed, and a probe was passed down from the alveolus into the wound. A small portion of the angle of the jaw was chiseled away, and the alveolus and the canal were thoroughly scraped. After the wound had been cleansed, a tampon of iodoform gauze was inserted into the alveolus, drainage-tubes were placed in the wound, and the edges were approximated with silk sutures. Primary union took place, and the patient made a rapid recovery. She gained over twenty-six pounds in weight within five weeks, and was now in good health.

*Case II.*—Thomas C., eighteen years old, consulted Dr. Murphy September 3, 1884, and stated that, on the previous Christmas, when he was living in Ireland, he had begun to suffer with severe toothache in the lower jaw, on the right side, which lasted about two months. Then a swelling appeared at the angle of the jaw, and the pain subsided. Since that time the swelling had enlarged somewhat, and he had had more or less pain. On examination, Dr. Murphy found a carious tooth, and the swelling showed well-marked fluctuation. It was as large as a pigeon's egg and situated below the jaw. There was a little induration of the surrounding parts. It was punctured, and about ten drops of thick, creamy pus of a consistence and color that attracted his attention, escaped, so that he made a microscopical examination of it and found that it contained radiating threads. Not being satisfied with this examination, he asked the patient to call again, which he did in two days. Pressure on the swelling then caused a small amount of pus to exude, and this was found to contain perfect actinomycetes. Specimens were shown to Dr. Fenger and Dr. Bridge, who recognized the parasite. The sinus was scraped out, and in ten days the parts were completely healed. The patient was seen four weeks later, and there was no evidence of the recurrence of the disease. About six weeks later the man again consulted the speaker in regard to a swelling in the same locality, somewhat larger than the first. It was opened, and a drainage-tube was inserted. The same characteristic pus was found, and it was examined by Dr. Fenger, Dr. Bridge, Dr. Belfield and Dr. Kerber, all of whom recognized actinomyces. The speaker had not seen the man since, but had learned that the opening had healed.

It would be seen from the summary Dr. Murphy, had given in his preliminary remarks, that in only one of the cases that had ended in recovery, had the disease begun in the lower jaw. Some of them appeared as diffuse phlegmons, some as chronic burrowing abscesses, and others as chronic suppuration of lymphatic glands, and, on opening the swellings, they were found to consist mostly of a mass of granulations, with but little pus, although Rosenbach had observed a moderate amount of matter in a few of his cases. Of the fatal cases, but two had had their origin in the lower jaw, the others originating in the internal organs, such as the lungs, the intestines, the oviducts, etc., and presenting no other symptoms than those of chronic suppurative inflammation, with little pain and no fever, but great emaciation. At the autopsies, the pus showed the characteristic granules, and the affected organs presented a honeycombed appearance, the spaces containing projecting masses of pale-yellow granulations and but very little pus.

The disease was very chronic in its course, its shortest duration, in the fatal cases, having been seven months, and the longest twenty months. The patients usually died of exhaustion or pyæmia. Various metastases might appear, either to the heart, the lungs, or the intestines. Considering the great space invaded, the comparative freedom from pain and fever was specially noteworthy. In the matter of diagnosis, it was important to notice that the disease usually had its origin in the lower jaw, whence it extended to the neck, the vertebræ, and the thoracic cavity; that it might have its origin in one of the internal organs; that, although the fistulæ extended deep and in all directions, their boundary was very sharp and the surrounding tissue but little disturbed, this being characteristic of the granulations; and, above all, that the only pathognomonic sign was the sulphur-colored granule, which, under the microscope, presented characteristic radiating threads with club-shaped ends.

As to the prognosis, the external forms of the disease showed seventy per cent. of recoveries, while the internal forms showed only eighteen per cent. The average mortality was sixty per cent. The treatment would depend upon the situation of the disease. When it was local, it was sufficient to open the mass and scrape and drain the cavity, or merely open it and wash it out with an antiseptic solution, as had been done by Rosenbach in his five successful cases. If the deposit was of any great extent, its radical removal was necessary. When the disease invaded the internal organs, we were completely at its mercy, and could only treat the symptoms as they arose.

*Discussion.*—Dr. Christian Fenger opened the discussion, during the progress of which, a number of microscopic specimens showing the actinomycetes were exhibited. Dr. Fenger had seen the two patients, whose histories had been related, but could add nothing to what had been said. Rosenbach's treatment might answer in slight cases, but the course of the disease was chronic, and it was often impossible to get at the deposit, even when its extension from the mouth was in the direction of the muscles of the neck, and especially was this the case when the swelling first formed at the angle of the jaw and the parasite got into the veins, resulting in metastases to the lungs or heart (and not on the outside of those organs). The fungi were too large to pass through the capillaries of the lungs, so that in case of such metastases, the patient was lost. If they got into the bronchi, or into the anterior or posterior mediastinum, not much could be done by medication, and they were beyond surgical treatment; hence a fatal result would follow. He would not hesitate to perform a radical operation wherever there was a sinus.

The President congratulated the author of the paper on the fact that he had been the first in this country to discover the disease in the human subject. He had been under the impression that the death-rate was about fifty per cent.

Dr. Tilly questioned the correctness of the diagnosis in the two cases that had been related. He had heard that an attempt had been made to inoculate a calf with the matter taken from one of the patients, and that it had failed. If that was the case, the diagnosis was not proved. Then, too, he would like to be assured that the specimens shown were really taken from these patients, and not from the bovine affection. He had seen the second patient after he had passed out of Dr. Murphy's hands, and recovery had followed within a fortnight after a tooth had been extracted. Why were not the diseased teeth extracted in both cases at the outset? He had no desire to throw cold water on the enthusiasm aroused by the alleged discovery, but he did not wish to see the Society take untenable ground.

Dr. Fenger replied that there was no longer any doubt as to the nature of the cases; there was the convincing proof of the microscope. The fungi were large, and not the contemptible micrococci. He cited a case in which the tooth was extracted at once, and yet the patient lingered for fourteen months, and died of metastases to the lungs and the vetebræ. Even if a patient seemed to have been cured, he should present himself to his physician every month for at least six months, that it might be seen if the disease showed any tendency to return.

Dr. W. T. Belfield testified to the accuracy of the diagnosis in the first case, which he himself had seen, and he had no doubt as to the correctness of Dr. Murphy's diagnosis in the second case; that gentleman knew what he was about, and had treated his patients correctly. He thought a misapprehension was being spread abroad with regard to the actinomycetes; they were not minute germs, but plants that could be seen with the naked eye. The cases reported by the writer of the paper were the first that had been observed and recorded accurately outside of Germany. Mr. Treves, of England, thought that he had recognized a case, but the microscope had disproved his diagnosis. Dr. Belfield then showed a large piece of the jaw-bone of a cow that had died of the disease. Having been permeated by the parasite, it was very soft, so that it could be cut with a knife. The cow's head had been swollen to nearly double its natural size, the enlargement being chiefly of the bone itself.

Dr. Tilley disclaimed any intention of throwing discredit on the microscope as a means of diagnosis, or of questioning Dr. Murphy's skill; but he was sorry that he had not seen the patients. The fact that the second one had recovered so quickly had given him the impression that the disease could not be so very dreadful. He would repeat that it was a pity the teeth had not been extracted early, and he thought Dr. Fenger's remarks were in favor of the view he himself took.

Dr. C. T. Parkes had seen the second patient after Dr. Lydston had extracted the tooth, but he had expressed no opinion as to the diagnosis. In all such cases the microscope ought certainly to be resorted to, in order to establish the diagnosis, but it should be remembered that an alveolar abscess might at times prove a very formidable affair.

Dr. Belfield said that, if these were not cases of actinomycosis, then scarlet fever was not scarlet fever on anybody's diagnosis. The disease spread by continuity, and perhaps might be analogous to anthrax. Anthrax was trifling if we cut it out at once, and so was actinomycosis, but, if the latter was allowed to spread to the neck, the chest, or the vertebræ, it became not only dangerous but incurable.

Dr. Murphy, replying to some of Dr. Tilley's criticisms, adds that one of the specimens from the case of the boy had been somewhat impaired by having been put into glycerine. In regard to the test by inoculation, both a calf and a horse had died within six weeks after having been inoculated with the pus. It made not a particle of difference what Dr. Tilley believed, or whether the teeth had been pulled out or not; the disease was certainly actinomycosis—the same disease, historically, clinically, and classically, as had been described by Rosenbach, and as such it had received proper surgical treatment by opening, drainage, etc. The "germs" were easy of recognition, and were herewith presented.

Dr. Tilley deprecated the introduction of any degree of animosity into the discussion, but it was still a pity, he thought, that the teeth had not been extracted in the early stage of the affection.

Dr. G. Newkirk thought that the limited number of cases reported cast some doubt upon the diagnosis, although it was true that successful inoculation upon the lower animals, together with the microscopical appearances, made it very strong. It would be a pity to extract a tooth for every swelling of the jaw, for ninety per cent. of them could be cured by simple measures.

Dr. Fenger remarked that it was an error to suppose that these cases were the first that had been reported outside of Germany, for an Italian physician had reported a case that had turned out to be pneumo-actinomycosis.

Dr. J. J. M. Angear called attention to the desirability of the members' knowing, with regard to the particular slides shown, which of them represented specimens from the cases reported, and which those that had been taken from animals.

Dr. William P. Verity corroborated Dr. Murphy's statements in regard to the fungi.

Dr. Belfield then showed a number of specimens, and compared them with the plates in Ponfick's monograph, which everybody could examine to his own satisfaction. With reference to alveolar abscesses, a fungus might be present in them, or the actinomyces might be found in them, but no one had ever looked for it, and he doubted if the pus in one case in a thousand, or one in ten thousand, was ever examined.

Dr. Parkes asked if the incipient symptoms of actinomycosis were as severe as those of alveolar abscess.

Dr. Belfield thought the actinomyces had never been found to have effected a lodgment in a healthy human being or a healthy animal, but it had been found in the mucus of the tonsils in hogs.

---

*Meeting of Jan. 5, 1885.*—The President, Dr. D. A. K. Steele, in the chair.

*The Late Dr. C. W. Chaffee.*—The Memorial Committee reported a preamble and resolutions eulogistic of the late Dr. Chaffee.

*Diphtheria; the Germ Theory and the Alcohol Treatment.*—Dr. G. H. Chapman read a paper on this subject in which he said that there was perhaps no known disease which had been so constantly and thoroughly discussed, and with regard to which we were still so much in the dark concerning trustworthy remedies, as diphtheria. Probably he would not be going astray if he stated that every physician was ready to adopt any method of treatment which offered a reasonable promise. Was the disease local in character, or did it involve the entire system ? Both theories had their advocates. Perhaps the most recent and best-founded theory was that given by Oertel, and confirmed by others, that the disease was first local and afterward developed into a general disease, and, moreover, that the infection was kept up by the local disease. The micrococci first attached themselves to the laryngeal mucous membrane, where they multiplied and produced inflammation of the adjacent tissues, and the membrane became filled with micrococci, which forced their way between the epithelial cells. The cells began to die and ulceration took place. The mouths of the lymphatics opened and the micrococci passed in. Early in the disease no micrococci were found in the blood, but, after the constitutional symptoms had become severe, masses of them would be found in the vessels, and the germ invaded the white blood corpuscles, which after a time would be found filled with micrococci. They would be found in the kidneys and in the capillaries, forcing their way between the muscular fibers of the heart and throughout the body, leading to destruction of tissue. Wood and Formad, of Philadelphia, had found that the attempt to inoculate animals with the poison derived from an ordinary case of diphtheria, as it appeared in that city, failed to produce the disease, but, when it was taken from subjects of a malignant type of the disease, they invariably succeeded in infecting the lower animals. They also produced diphtheria with micrococci cultivated in soups, even to the fourth or fifth generation.

They further found in ordinary saliva a micrococcus which could not be distinguished from that found in the membrane from the most malignant diphtheria, except that with those they could not produce the disease; and also that the action of the poison was in direct proportion to the virulence of the of the epidemic. The former writer thought that diphtheria was produced by micrococci that were present in everybody's mouth. These were some of the conclusions of those who had spent years in the investigation of this subject, with abundance of material at their disposal.

Granting that the deductions of these investigations were correct, there must be conditions for the rapid reproduction of the germs which were not yet understood; and might it not be that they found in the degenerated or septic condition of the fluids and tissues a good field for growth and development in an active form, and still were not the primary cause of diphtheria? But, whether the disease was purely local or general in its origin, whether micrococci were or were not the primary cause of its development, the stubborn fact remained that, in nine cases out of ten, when the physician was first summoned, he found abundant evidence of systemic disturbance, varying in intensity from simple circulatory excitement to the most profound prostration, like that of septicæmia.

A severe form of this disease was manifested in South Chicago during the autumn and winter of 1881–'82. In the first six weeks of the epidemic, the writer treated seven cases, with two deaths and one protracted recovery out of three malignant cases. The treatment consisted, *externally*, in the free use of camphorated oil with turpentine, and applying to the membrane every four or six hours, with a camel's-hair brush, a mixture of a drachm of carbolic acid, three fluidrachms of liquor ferri sub-sulphatis, and half a fluidounce of glycerine. The throat was gargled with a saturated aqueous solution of chlorate of potassium. *Internally*, tincture of chloride of iron and quinine were given, in doses suitable to the age of the patient, giving to a child, eight years of age, two grains of quinine and six drops of tincture of chloride of iron every three hours. In one of the cases that seemed to be malignant, but ended in recovery, dry sulphur was used after the fourth day, blown freely into the throat and nasal cavities after cleansing the parts with chlorate of potassium solution. There had been, up to this time, in all, about twenty cases in the immediate vicinity, with twelve deaths. Other physicians had had no better success in combating the disease. At this time the writer's attention was called to the free use of alcohol in this disease, by various publications, from which it appeared that alcohol was one of the best agents for destroying the micrococci or rendering them inert; and he determined to give it a fair trial, using it diluted, with chlorate of potassium as a gargle, especially using the latter remedy before the patient took either medicine, food, or drink. Dry sulphur was also used in the throat and nasal cavities, with the internal use of whisky-sling or milk-punch, as hot and strong as could be borne, and only to be limited by the amount which could be tolerated by the patient without producing intoxication, along with other agents, such as diuretics, antifebrile remedies, etc., as each case seemed to require.

As a result of this trial, his succeeding twenty-eight cases were carried to a successful termination with one exception, and in none of them was a brush or probang used.

The alcohol in every case could be used very strong; it would almost invariably cause the membrane to shrivel up like burning leather, and within thirty-six to forty hours every trace of membrane disappeared. Tincture of veratrum viride and deodorized tincture of opium were given in some cases for fever during the congestive stage only.

During the past two years the author had relied on the liquor-ferri-subsulphatis-and-carbolic-acid mixture applied to the throat with a camel's-hair brush twice or thrice a day, or still more frequently. He pursued this plan instead of blowing in sulphur with a bellows or through a goose-quill. The sheet-anchor, however, was alcohol in every stage of the disease, and, from his experience, he was led to think we could secure much better results with it than by the harsher methods o treatment.

[Here the author gave brief accounts of a number of illustrative cases.]

As before stated, only one patient of the twenty-eight treated with alcohol, was lost; a girl eleven years of age, of nervous temperament, who, six months previously, had had a severe attack of typhoid fever, from which she had not yet fully recovered Consequently when she was attacked by diphtheria of a severe naso-pharyngeal character, it was difficult to maintain her strength, and she died on the eighteenth day, from paralysis of the heart.

In July, 1882, the writer attended three cases of croupous diphtheria. The first two do not require any special mention. The third case of this series was under the care of another physician for two days before he was called. It seemed impossible for life to be prolonged an hour. However, a teaspoonful of a mixture of a grain of calomel, five minims of tincture of aconite-root, and four ounces of water was ordered to be given every hour. Milk-punch and whisky-sling also to be given frequently. Instructions were given that, if the child lived until evening, he should be sent for. The child lived, and the treatment was continued. Upon visiting the little patient the following morning, he was greatly surprised at seeing the child running about the room dragging a broom and in high glee. It would be seen that great relief followed in this case within twelve hours. Doubtless the calomel had had much to do toward bringing this about.

*Discussion.*—Dr. G. C. Paoli thought the author was too prone to follow out routine treatment in diphtheria, whereas on such form could be laid down in this disease. Why? In malignant cases the patient might live only two, three, or at most five days, and this was not the fault of the physician. However, alcohol was no new remedy in these cases. It had been used forty years, chiefly, though, in the form of wine. Alcohol might be given in these cases to destroy fungi or bacilli, on the same theory that it was given as an antidote for snake-poison. But the question arose, would alcohol save patients where there was fibrinous exudation upon the tonsils, pharynx, and other parts; where it was of an ashy-gray appearance; where the maxillary and cervical glands were involved; where there was a dusky hue of the face, with cyanotic lips, and feeble heart and pulse, fœtid breath, hyperæmia of the brain, etc.? He would hesitate, on general principles, about giving the remedy. Instead, the first thing that should be done was to use antiseptics, such as oil of eucalyptus, which would destroy the bacilli. For young children, he would use it with an atomizer, for they could not gargle. Where there was ulceration of the naso-pharyngeal space, hæmorrhage might set in, and nothing would check it. Internally we should give tincture of iodine, in water, for a child five years of age, four drops every hour; and apply any of the well-known antiseptics with a probe or brush to the throat. It was of no use to give them quinine and tincture of iron, for these remedies they could not take, and, besides, they interfered with the action of the kidneys and liver

Dr. R. Tilley desired to add his testimony to the special value of tincture of iodine in these cases, although no one remedy could be used in all cases. He also thought corrosive sublimate was beneficial, and he placed great reliance upon it. But he preferred the use of hot baths in the early stage of this disease as facilitating profuse perspiration instead of using veratrum viride. He was satisfied that bacteria were a causative agent of this disease.

Dr. J. J. M. Angear said that the microscopists had settled it that micro-organisms or microbes were a cause of diphtheria. Alcohol, as was well-known, destroyed vegetable growths. A fifteen-per-cent. dilution would prostrate or paralyze them, and, if its use was continued, would kill them. He therefore thought it a good local application. But these germs flourished best in a high temperature. We could not get alcohol as strong as a fifteen-per cent. dilution to circulate in the blood. Therefore, how did it kill them when the blood contained them? Then, too, why administer any alcohol and veratrum? Did not these remedies antagonize each other? There was harmony between the germ theory and the local use of alcohol. But he should think cold applications were preferable to hot drinks or to hot baths, for the reason that germs flourished better under a high temperature.

Dr. A. Leigh had found, in treating more than a hundred cases during the past four years, that, of all agents *per se*, hot or cold, there was none better than a concentrated or dilute preparation of the perchloride of iron. But in really malignant cases no treatment was of any avail. Alcohol, hot or cold, was a good cardiac stimulant, but quinine would do better work. Tincture of iron would produce contraction or hardening and shriveling of the membrane. There was no doubt that micrococci were present in the throat in this disease. Therefore, if the cases were seen early, and the iron was applied, benefit would be derived, except where the case was profoundly malignant. Tincture of aconite was superior to veratrum to bring about free diaphoresis.

Dr. H. J. Reynolds had come to the conclusion that there were no remedies that had a specific effect on diphtheria. The most that could be done was to rely upon supporting measures to help the patient along until the disease ran out. If the membrane entered the larynx, he would advise inhalation of steam. He thought no benefit would be derived from alcohol or veratrum. When the disease was malignant at the onset, no treatment was beneficial.

Dr. A. Goldspoon had treated a hundred cases of diphtheria during the last two years. He had seen great constitutional disturbance present when the local signs were quite insignificant, and cited a case of this kind occurring to a little girl that died in ten hours from the time she was attacked. The system was so depressed that she died of heart failure. In such cases it would be reprehensible to administer aconite or veratrum. To reduce the temperature, he relied upon quinine. Alcohol could not be given sufficiently concentrated to act through the circulation as a germicide, although it might do good locally.

Dr. Paoli said that when diphtheria appeared in countries, and at a season when the temperature was very low, it was equally destructive. Alcohol might prove beneficial in some cases that were peculiarly asthenic.

The President recited an epitome of Morrell Mackenzie's views upon the pathology and treatment of diphtheria.

Dr. W. H. Wilson thought that we could not give sufficient whisky to a child having diphtheria to intoxicate it. Recently he had treated six cases of the disease in children, five of whom recovered, and to these he gave large quantities of alcohol. In the other case, the parents (on moral grounds) would not permit him to give the child whisky, and he was confident that, had he been permitted to do so, this child also would have recovered.

Dr. Chapman conceded the impossibility of producing intoxication in a patient having diphtheria. He maintained that by treating cases in this way, paralyses were made less liable to follow. He was sure it also quieted the cerebral symptoms such as delirium, and did not increase hyperæmia of the brain. Regarding the administration of veratrum and alcohol alternately, he did this during the first or congestive stage, or until perspiration was well established. We should begin the use of alcohol as early as we were sure of our diagnosis. By so doing, the fungi were destroyed and we prevented their entering the blood-vessels. If a few did enter the circulation, it was not so bad as if they passed in in swarms.

---

CHICAGO GYNECOLOGICAL SOCIETY.

